# Mastoid Inflammation with a Report of Two Interesting Cases*Read by title before the Southern Section of the American Otological, Rhinological and Laryngological Association, Atlanta, March 28th.

**Published:** 1898-05

**Authors:** T. E. Mitchell

**Affiliations:** Columbus, Ga.


### MASTOID INFLAMMATION WITH A REPORT OF TWO INTERESTING CASES.*

BY T. E. MITCHELL, M.D.,
COLUMBUS, GA.

As an apology for my remarks on mastoid inflammation I desire to say that it is a subject which merits our careful consideration for the reason that it is always a grave disease, and though relatively infrequent, when present demands prompt and rational therapeutic agents. In medico-surgical parlance the mastoid process consists of that part of the temporal bone immediately posterior to the attachment of the auricle. Ordinarily it contains a honey-

* Read by title before the Southern Section of the American Otological, Rhinological and Laryngological Association, Atlanta, March 28th.

combed structure of bone with cells variable in size, shape and number in different individuals, and in the two mastoids of the same individual. One of these, the mastoid antrum, is always present and sometimes, though rarely, exists alone. These mastoid cells are lined by a continuation of the tympanic mucous membrane. They intercommunicate with each other and by way of the antrum with the tympanum, and, like the latter cavity, contain air, hence the name pneumatic cells. This osseous structure is in relation with the middle cranial fossa above and the external auditory canal in front, from which it is separated by thin layers of bone. The sigmoid portion of the groove for the lateral sinus lies internal to the cells and usually to their posterior, though it is not constant in this respect. The depth of the antrum from the cortical layer varies from one-half to seven-eighths of an inch.

Except in rare instances mastoid inflammation is secondary to a suppurative inflammation of the middle ear. Because of the very intimate anatomical relation between the tympanum and mastoid cells it is difficult to understand how an inflammation of any magnitude can exist in the former without there being at the same time a similar pathological condition in the latter; and yet in the vast majority of instances in which there is a discharge of pus from the middle ear no coexistent symptoms referable to the mastoid are present.

Mastoiditis may exist in various degrees of intensity, from a mere congestion of the mucous membrane lining the cells to a condition in which the trabeculæ as well as the interspaces are obliterated and replaced by a homogeneous mass of pus and inflammatory débris.

Probably no two cases of mastoid disease are exactly alike, and yet in the vast majority of instances the course and symptoms are sufficiently characteristic to enable one to make a reasonably certain diagnosis. The earliest, most constant and characteristic symptoms are pain in the post-auricular region and tenderness on deep pressure over the antrum. More rarely these are accompanied by constitutional disturbances, local edema and redness. If an early diagnosis be followed by judicious treatment, the inflammation may be checked in its early stages and the parts restored to their normal condition. This is more often true when the disease complicates an acute middle ear inflammation. On the other hand, one stage of the pathological process may follow another to the death of the parts and the formation of pus.

A pouting, sacculated condition of the postero-superior cutaneous lining of the external auditory canal close to the drum membrane, together with redness and tenderness over the mastoid antrum, are pathognomonic signs of pus in the cells.

So soon as symptoms of mastoid congestion are manifest, absolute rest in bed should be ordered, together with a saline cathartic and liquid diet. Cold in the form of the Leiter coil or aural ice-bag should be kept on the mastoid, and the ear frequently syringed with hot water from a fountain syringe. At the same time free drainage through an artificial opening in the drum-head should be encouraged. This line of practice will ameliorate the symptoms in the majority of cases, and in a fair per cent. will, if applied early and efficiently, abort the trouble. If, however, marked improvement is not had in forty-eight to seventy-two hours the probabilities are that symptoms demanding an operation will be manifest. The cells should be opened by way of the cortex and all detritus removed as soon as possible after the formation of pus which, if not removed, will seek an outlet in the way of least resistance, and should this be in the direction of the middle cranial fossa or backward towards the lateral sinus, a fatal meningitis or thrombosis would likely result. Fortunately the pus usually burrows its way through the cortex along the tract of a mastoid emissary vein.

Since the time of Sir William Wilde an operation bearing his name has been more or less practiced for aborting mastoid inflammation. It consists of a vertical incision parallel to the attachment of the auricle down to and through the mastoid periosteum. When we call to mind that the beginning of a mastoid inflammation is in the antrum with an overlying dense layer of bone, variable in thickness from one-fourth to seven-eighths of an inch, the Wilde incision to my mind is as unwise and unsurgical as it is inefficient as a therapeutic agent. With free drainage through the drum, frequent syringing with hot water, together with the anodyne effect of cold to the mastoid and absolute rest in bed, many cases will recover.

A case in point: A. M. C., aged forty-six, bridge-builder, while sleeping under a tent contracted a severe cold followed by acute inflammation of both middle ears. When first seen by me both drums were very red and bulging. Both mastoids were red, slightly swollen, exquisitely tender with characteristic pains and considerable constitutional disturbance. Incision of drums was refused. Ordered the patient to bed and prescribed as indicated above. In twenty-four hours the patient was fairly comfortable, but the right drum had ruptured. Symptoms continued to improve and on the fourth day applications to the mastoid were discontinued. Hot water from a fountain syringe and inflations constituted the remaining treatment. The perforation healed in two weeks, and although the patient passed from under my observation before the hearing was entirely restored, it was practically so when I last saw him.

I believe that pus beneath the soft parts overlying the mastoid cortex during a mastoiditis always forms in the cells within and makes its way out either by way of the tympanum along the superior canal wall or through a fistulous tract in the cortex. It is only in these cases that the Wilde incision is indicated, and since the seat of trouble is within the mastoid, good surgery would suggest that the use of the knife be only preliminary to a resort to the chisel. The operation *per se*, is not a dangerous one, and when properly done the patient's prospects for a speedy recovery are infinitely better than when drainage is so imperfect as is the case after the incision alone.

CASE I.—Mr. M. B., aged thirty-three, of robust physique without any history of a previous ear trouble, contracted cold from exposure to rain July 7, 1896. As a result he had an acute suppurative inflammation of the right ear. A spontaneous rupture of the drum-head and consequent discharge of pus stopped under treatment in twelve days. Hearing at this time equaled watch in contact, which was increased to two inches after inflation. Patient resumed his vocation as traveling salesman, reporting at my office once a week with the uniform statement that he suffered with pain of a neuralgic type in the occipito-parietal region of the affected side. This state of affairs continued for nearly six months till January 3, 1897, during which time there was absolutely no pain, redness, swelling or tenderness over the mastoid. Dr. A. W. Calhoun, of Atlanta, saw the patient at intervals during this time, and agreed with me that the mastoid was not diseased and that the pains were neuralgic with an obscure origin, probably malarial; but the negative results of our therapeutics and the subsequent history of the case proved the fallacy of our diagnosis.

January 3, 1897.—A circumscribed swelling over the antrum has made its appearance during the past forty-eight hours and with it considerable tenderness on pressure. Under local anesthesia an incision was made in the swelling, evacuating more or less pus, and drainage kept up for two weeks.

On January 19, very little or no relief having followed the less radical operation, I advised an opening of the cells, which I did under ether. A fistulous tract was enlarged and the cells were found to be one large cavity filled with creamy pus and inflammatory débris, the trabeculæ having been obliterated. This was all removed and the bone well curetted, not, however, without an unavoidable opening of the lateral sinus; but since this occurred just at the close of the operation, the hemorrhage was stopped with an iodoform tampon followed by the usual toilet, after which the patient was put to bed in good condition.

January 20, 9 A.M.—Patient reacted well, no pain, temperature normal, pulse 78. At 2 P.M. pulse 92, temperature 102, which reached 103 by 10 o'clock.

January 21, 9 A.M.—Patient resting well, no pain, pulse 80, temperature 101. At 10 P.M. general condition the same, pulse 86, temperature 102.

January 22, 9 A.M.—Resting well, pulse 86, temperature 102. Original dressing removed. At 2 P.M. pulse 94, temperature 103½, which fell to 102 by 10 o'clock.

January 23, 9 A.M.—Patient comfortable, pulse 75, temperature 99⅘. At 7 P.M pulse was 85 and temperature 101⅕. Appetite, which has been poor since the operation, is now good.

January 24, 9 A.M.—Pulse 75, temperature 99.

January 25.—Patient comfortable, no pain, appetite good, pulse and temperature normal. Further history of the case was uneventful, and on February 28th, forty days after the operation, the wound was entirely healed, the patient was dismissed well, with his hearing quite as acute as it ever was. The interesting features in this case are:

(1) The entire absence of any symptoms indicative of mastoiditis during the suppurative stage of the middle ear inflammation. (2) The lapse of time between the active middle ear inflammation and the manifestation of pus in the cells. (3) The entire absence of any and all symptoms referable to the mastoid region during the formation of pus therein. (4) The remote situation of the pain in the occipito-parietal region.

Case II.—Mr. S. P., aged thirty-two, bookkeeper, when a child had frequent attacks of earache, but does not remember that there was ever a discharge from the canal. In August of 1897 he had malarial fever from which he recovered in two weeks. About the time of convalescence he developed an earache and a coincident mastoid tenderness on the left side. This continued for about ten days, when all pain and tenderness disappeared leaving only a stuffy feeling in the ear with impaired function. At no time was the pain in the ear or mastoid severe enough to interfere either with his work by day or rest at night. On the 5th of December following, the mastoid became again tender and painful. This continued to an extent not sufficient to interfere with his duties or rest for twelve days, when, on December 17th, he consulted me for the first time. Upon examination I found the drum intact, the mastoid red, swollen and tender on pressure, with no great amount of pain and no constitutional disturbance. I ordered the patient to bed, gave a cathartic and kept applications of ice to the mastoid for forty-eight hours. During this time the patient was entirely comfortable, but the condition of the soft parts over the mastoid suggested pus within, consequently a radical operation was advised. This, however, was refused till the 23d when, under ether narcosis, the cells were opened. A long incision was made extending from the tip of the mastoid below to a point above the auricle down to and through the periosteum, which being scraped aside disclosed a fistulous opening leading to the antrum. The entire cortex down to the tip being soft from decay was easily chiseled away, exposing the interior of the mastoid, which was one large cavity filled with thick pus and granulation tissue. This was all removed and the bone well scraped with a sharp spoon till it appeared clean and glistening, after which an iodoform dressing was applied and the patient put to bed.

December 24, 9 A.M.—Patient reacted nicely, ate supper, slept well during the night and is entirely comfortable with the pulse 70 and temperature normal. From this date the patient made an uninterrupted recovery, there being at no time any pain, fever or acceleration of pulse. The hearing rapidly improved from a watch in contact before the operation, to its original acuteness. The patient was dismissed in seventy days with the opening entirely closed and a scar satisfactorily small.

The interesting features of the latter case are: (1) The remission of all symptoms except the impaired hearing, from September to December. (2) The development of a mastoid abscess with an intact drum and no evidence of suppuration in the middle ear. (3) The entire absence of any constitutional disturbance and the very mild character of the pain during the inflammatory process that eventuated in so much destruction of tissue and pus formation.

